# Ontology patterns for the representation of quality changes of cells in time

**DOI:** 10.1186/s13326-019-0206-4

**Published:** 2019-10-16

**Authors:** Patryk Burek, Nico Scherf, Heinrich Herre

**Affiliations:** 1 Institute for Medical Informatics, Statistics and Epidemiology, University of Leipzig, Haertelstr. 16-18, 04107 Leipzig, Germany; 2 Institute of Computer Science, Faculty of Mathematics, Physics and Computer Science, Marii Curie-Sklodowskiej University, pl. Marii Curie-Sklodowskiej 5, 20-031 Lublin, Poland; 3 Max Planck Institute for Human Cognitive and Brain Sciences, Stephanstr. 1a, 04103 Leipzig, Germany; 4 Max Planck Institute of Molecular Cell Biology and Genetics, Pfotenhauerstr. 108, 01307 Dresden, Germany; 5 Carl Gustav Carus Faculty of Medicine, Institute for Medical Informatics and Biometry, TU Dresden, Fetscherstr. 74, 01307 Dresden, Germany

**Keywords:** Ontology, Design patterns, Cell tracking, Web ontology language

## Abstract

**Background:**

Cell tracking experiments, based on time-lapse microscopy, have become an important tool in biomedical research. The goal is the reconstruction of cell migration patterns, shape and state changes, and, comprehensive genealogical information from these data. This information can be used to develop process models of cellular dynamics. However, so far there has been no structured, standardized way of annotating and storing the tracking results, which is critical for comparative analysis and data integration. The key requirement to be satisfied by an ontology is the representation of a cell’s change over time. Unfortunately, popular ontology languages, such as Web Ontology Language (OWL), have limitations for the representation of temporal information. The current paper addresses the fundamental problem of modeling changes of qualities over time in biomedical ontologies specified in OWL.

**Results:**

The presented analysis is a result of the lessons learned during the development of an ontology, intended for the annotation of cell tracking experiments. We present, discuss and evaluate various representation patterns for specifying cell changes in time. In particular, we discuss two patterns of temporally changing information: n-ary relation reification and 4d fluents. These representation schemes are formalized within the ontology language OWL and are aimed at the support for annotation of cell tracking experiments. We analyze the performance of each pattern with respect to standard criteria used in software engineering and data modeling, i.e. simplicity, scalability, extensibility and adequacy. We further discuss benefits, drawbacks, and the underlying design choices of each approach.

**Conclusions:**

We demonstrate that patterns perform differently depending on the temporal distribution of modeled information. The optimal model can be constructed by combining two competitive approaches. Thus, we demonstrate that both reification and 4d fluents patterns can work hand in hand in a single ontology. Additionally, we have found that 4d fluents can be reconstructed by two patterns well known in the computer science community, i.e. state modeling and actor-role pattern.

## Background

Life is a complex, hierarchical and *dynamic* process [1]: it is a hallmark of all living systems that they change over time. This is obvious during development, regeneration, or disease; but even under homeostatic conditions living matter is in a dynamic equilibrium; an example is the constant turnover in the hematopoietic system to maintain a certain number of blood cells. Thus, a deeper understanding of basic biological principles requires us to resolve the system’s spatial and temporal structures [2]. Over the past decades, advances in biomedical imaging, experimental procedures, and computational analysis led to the establishment of time-lapse microscopy that allowed us to study the spatio-temporal organization of tissues, organs, or whole animals at the cellular level [3, 4].

Time-lapse microscopy has become a fundamental experimental tool in biomedical research. The goal is to reconstruct migration patterns, shape changes, changes in protein expression and, eventually, comprehensive genealogical information [5, 6] from the data. However, the analysis of the resulting videos has become a major bottleneck: manual analysis can be done on short sequences with few cells, but it is practically infeasible for large-scale, systematic experiments. Consequently, the development of computational tools for *cell tracking*, either fully or partly automated, is a vital field of research in image analysis [7–9]. However, what is still largely missing is a structured, standardized way of annotating and storing the tracking results. But this is exactly what we need in the future to build systematic databases of cell tracking experiments and to mine, infer and compare the inherent biological information.

A few steps have been taken in this direction: in [10–12] we reported on our work in progress on the framework for annotating results of experiments and simulations in stem cell biology. The core component of the framework is a Cell Tracking Ontology (CTO) formalized in the Web Ontology Language (OWL) [13], which enables the annotation of time-lapse experiments.

Typically, the information about a cell’s history is organized into pedigree-like data structures called *cellular genealogies* [14]. In such a genealogy the root represents the founder cell and its progeny is arranged in the branches of the tree, with branching events representing cell division. Here, a cell is perceived as a spatially and temporally extended object. The existence of a cell is temporally restricted by its birth (the division of the mother cell) and either its death (apoptosis) or terminal division (mitosis), yielding two daughter cells of the next generation [10]. The observed cells themselves are dynamic entities, i.e. they can change their shape, their position (migration), or their internal state (differentiation). Therefore, the key requirement for an ontology of cellular genealogies is the representation of changes over time in individual cells, such as, for instance, the change of a cell from a round to an elongated shape.

Unfortunately, representing temporal information is a serious problem appearing across numerous areas of information modeling, including data modeling and relational database design [15] as well as the semantic web languages typically used for ontology modeling, such as Resource Description Framework (RDF) [16], Web Ontology Language (OWL), or the Description Logics underlying OWL. One problem originates from the lack of direct support for n-ary relationships, which in turn limits the capabilities for representing temporally indexed information.

One possible approach to overcoming this limitation is the extension of these languages so that they can express temporal information. For instance, in the area of description logics numerous approaches have been proposed to incorporate time into the logic model [17–19]. Unfortunately, as discussed in [20], temporal logics still have problems with representing temporally changing information, as they are geared towards synchronic relationships, not diachronic ones. In the field of RDF the incorporation of temporal information and temporal reasoning has been proposed by [21] using the so-called temporal RDF graphs or a query and storage syntax [22]. For OWL, [23] proposed an extension for representing dynamic entities using a four-dimensional (4d) model.

An alternative to language extensions, and one more relevant for the development of CTO, is a solution on the user level without any need to modify the language itself. Along these lines, numerous patterns have been proposed [24], and two strategies are of particular interest: *reification of n-ary relations* and *4d fluents*.

The former strategy is rather straightforward: an n-ary relationship is represented by introducing an additional model element, the so-called *reified entity*. This approach is well known in many areas of information modeling, e.g. Associative Entities in Entity-Relationship-Diagrams (ERD)[25], Intersection Tables in SQL [26], or Association Classes in Unified Modeling Language (UML) [27]. This strategy has also been suggested for OWL [28].

The alternative approach originates from the philosophical theory of four-dimensionalism [29], where entities are considered as the so-called 4d worms, which can be sliced into temporal parts. Different variants of the 4d fluents pattern have been introduced in literature, among others by [24, 30–32], yet all have in common the same underlying principle, which, analogously to the reification strategy, introduces to the model an additional entity (or entities) representing temporal information. However, in contrast to reification, the introduced entity does not represent a reified relationship but a temporal part/slice of a modeled entity.

In the current paper we take a closer look at both patterns and their relevance to modeling the dynamic change of information in biomedical ontologies such as CTO. We focus primarily on the application of these patterns for constructing new ontologies from scratch. It should be noted that our goal is neither to address other related issues, such as time representation and temporal reasoning, nor the extension of existent OWL domain ontologies, as it has been presented e.g. in [33, 34].

Although we focus on the use-case of cell tracking experiments, the problem is generic and has to be addressed by ontology engineers in different domains of the biomedical field. In contrast to many overviews of the discussed problem, which typically conduct their analyses on single isolated temporal information, we focus on a dynamically changing web of information. We demonstrate that the patterns perform differently depending on the temporal distribution of the information. We further demonstrate that a 4d approach can be re-constructed using other well known patterns not requiring the introduction of 4-dimensionalism. We conduct our analysis discussing the benefits and drawbacks of each pattern with respect to common criteria for software systems and information modeling, i.e. scalability, extensibility and adequacy.

### Cell tracking

**Fig. 1:**
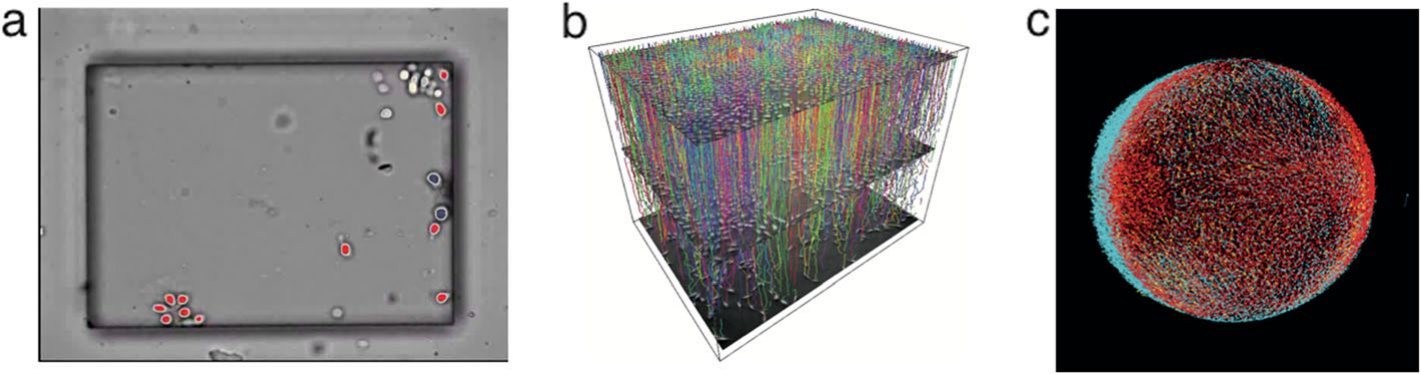
Examples of cell tracking data: (**a**) In vitro tracking of an initially small number of cells. (**b**) In vitro tracking of a fast expanding culture of pancreatic cells (200 images, 4600 cells in total). Resulting trajectories and genealogies are shown in a space-time plot. (**c**) In vivo tracking of early zebrafish development over several hours (400 images, 10,000 cells per image)

**Fig. 2:**
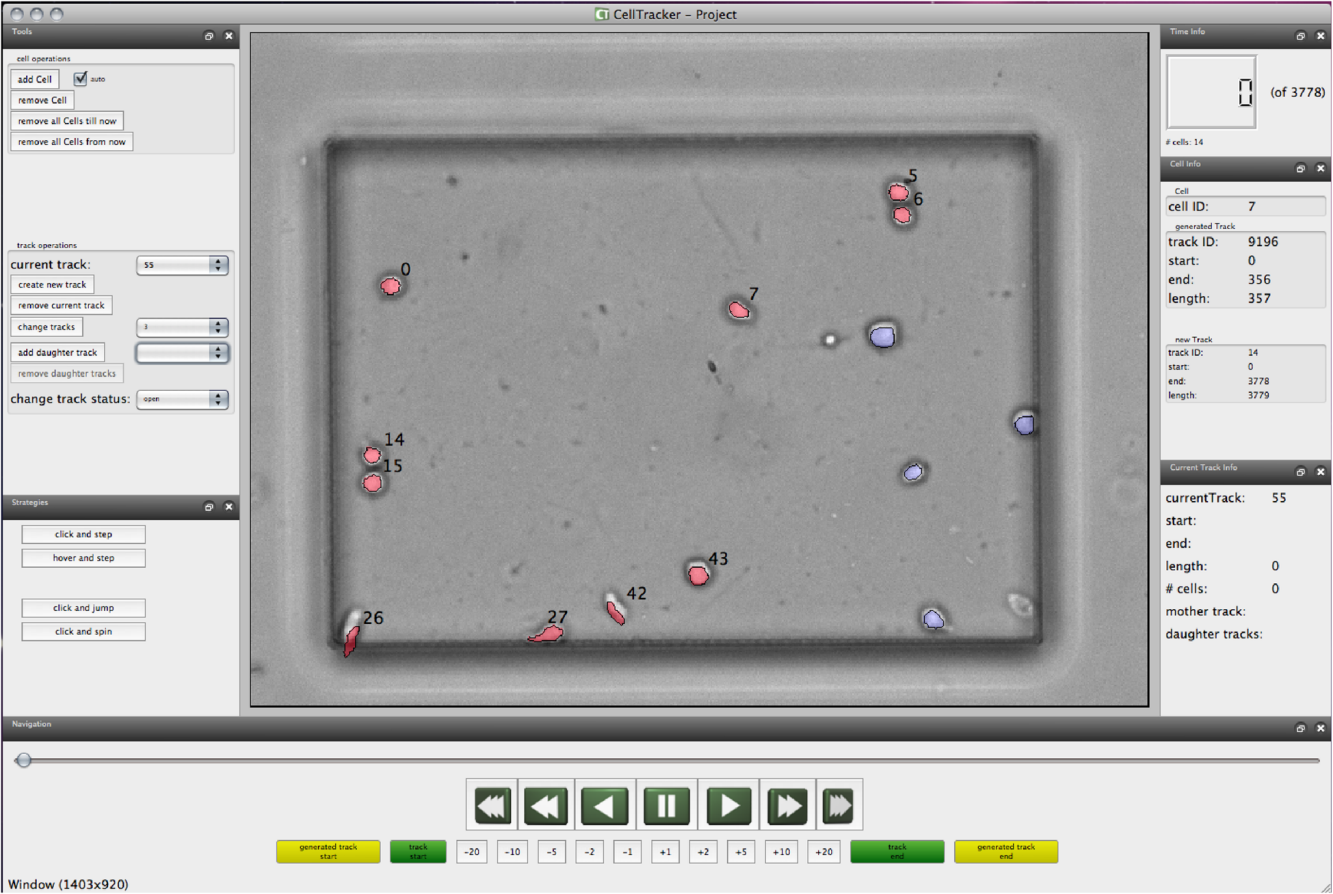
Screenshot: An example showing a software for manual correction and annotation of cell tracking experiments as described in [11]

To get an idea of the Cell Tracking Ontology, it is useful to sketch some aspects of typical cell tracking experiments. Figure 1 demonstrates examples from the variety of image sequence data acquired via time-lapse microscopy, which can be either two-dimensional (e.g. in vitro experiments using traditional wide-field microscopy) or three-dimensional volumes (e.g. in vivo imaging using fluorescence microscopy) over time. The data are corrected, annotated and analyzed with the help of software tools such as [11, 35–37]. The tool from [11] is shown as an example in Fig. 2. A single experiment can span from a few hours [38] to several days or even weeks [39, 40], depending on the frame rate (i.e. the time between two consecutive snapshots). This yields a few hundreds up to thousands of images (or image volumes) per experiment. The number of cells observed in each image varies with biological applications: from tens of cells in in vitro stem cell assays [39] to tens of thousands of cells in developmental studies [4, 8, 41]. That is, the total number of individual observations (snapshots of a cell in time) in a database will lie somewhere between 10,000 and 500,000 for typical studies but can easily reach a few million.

For biological studies, a number of cellular features should be recorded and properly stored in the database. There are various qualities of interest which are associated with a snapshot of a single cell; these can be classified and systematized within a top level ontology of data, being a part of GFO [42]. Examples of such snapshot qualities are:

- *Cell position*: typically given as the centroid of the cell in Cartesian coordinates as a vector [*x*, *y*] in 2D or [*x*, *y*, *z*] in 3D,
- *Cell shape*: either given as a mask (a set of spatial grid elements (voxels) occupied by the cell), as a polygonal outline, or in an abstract representation (e.g. as an oriented ellipsoid),
- *Cell dimensions*: e.g. the area or volume occupied by the cell,
- *Cell state*: usually measured as the concentration, or the presence/absence of certain gene products (RNA or protein) found in particular cell types,
- Depending on the research question additional features could be of interest, e.g. cell polarity, orientation, or intracellular features.

At the level of cell trajectories, the following features would be of interest:

- *Migration*: the speed and direction of cell movement,
- *Deformation*: changes in cell shape,
- *Mitosis*: occurrence of cell division,
- *Apoptosis*: occurrence of cell death,
- *Differentiation*: changes in cell type reflected by changes of other features, such as genetic markers.

Finally, using the information gathered in complete genealogies a number of aspects can be analyzed:

- *Topology*: overall structure of the pedigree and its sub-trees,
- *Cell cycle kinetics*: distribution of cellular life-times,
- *Fate maps*: the identification of sub-populations of cells that give rise to certain structures of interest (e.g. tracing the origins of specific organs),
- *General structure of sub-populations*: the distribution of different features (e.g. cell fate) within the genealogy,
- *Inter-cellular communication*: the influence of cell behavior by other cells within its spatial and temporal neighborhood.

### Preliminaries: terminological clarifications and problem statement

We do not make many ontological restrictions on the top level categories used for the development of an ontology. The broad spectrum of top level categories, which can be utilized for knowledge representation in general and for ontology development in particular, can be found in literature [43, 44].

We do not want to limit the analysis to any of those ontologies, instead we only make a few common-sense assumptions, keeping in mind that even those presuppose some ontological commitments, hence ontology cannot be escaped:

- Entities such as cells endure through time spans called their lifetimes. We call these *Objects* (Obj).
- Objects such as cells possess certain characteristics describing them. Those characteristics, called in the current paper *Qualities* (Q), are expressed in natural and artificial languages by means of syntactic elements such as adjectives/adverbs or attributes/properties, respectively (p. 30, [45]). Typical qualities of cells are e.g. round shape or a specific location.
- A quality such as an oval shape can be predicated upon an object in a sense that we can say that the object has that quality as e.g. a cell has an oval shape. In the current paper we will call such assignments *Quality Assignments* (QA).
- A particular QA can change over time. For instance, the shape of a cell can change, i.e. shape quality at two different time points may differ. We refer to all types of entities as *Time Entities* (TE). Those are e.g. intervals and time points.^1^ A complete analysis of the current time-ontologies is presented in [46], which also includes a comparison between GFO-Time and Allen’s theory of temporal intervals [47]. Allen’s theory can be reconstructed within GFO-Time, though the converse is not possible. GFO-Time provides a coincidence-relation between time-boundaries which allows to model discrete changes, if needed..

Putting the above together allows us to interpret the sentence “a cell has an oval shape at time *t*_1_ ” as follows: a cell *c* is an object, a round shape *o* is a quality and *t*_1_ is a time entity indicating the time-extent of quality assignment of *o* to *c*.

Based on the above assumptions and terminological clarifications the problem addressed in the current paper can now be formulated as follows: How to model the change of an object’s quality assignments over time in OWL? E.g. how to represent a change of a cell’s shape from round at time *t*_1_ to elongated at time *t*_2_ ?

We believe that in ontology development, as it is recognized in software engineering [48], there is no uniquely determined approach which is optimal in all contexts. For this reason, the goal of the current paper is not to provide some ultimate template for modeling a change of an object’s qualities, but instead to review possible patterns and verify them against our specific use case of developing CTO.

### Problem statement exemplified

**Fig. 3:**
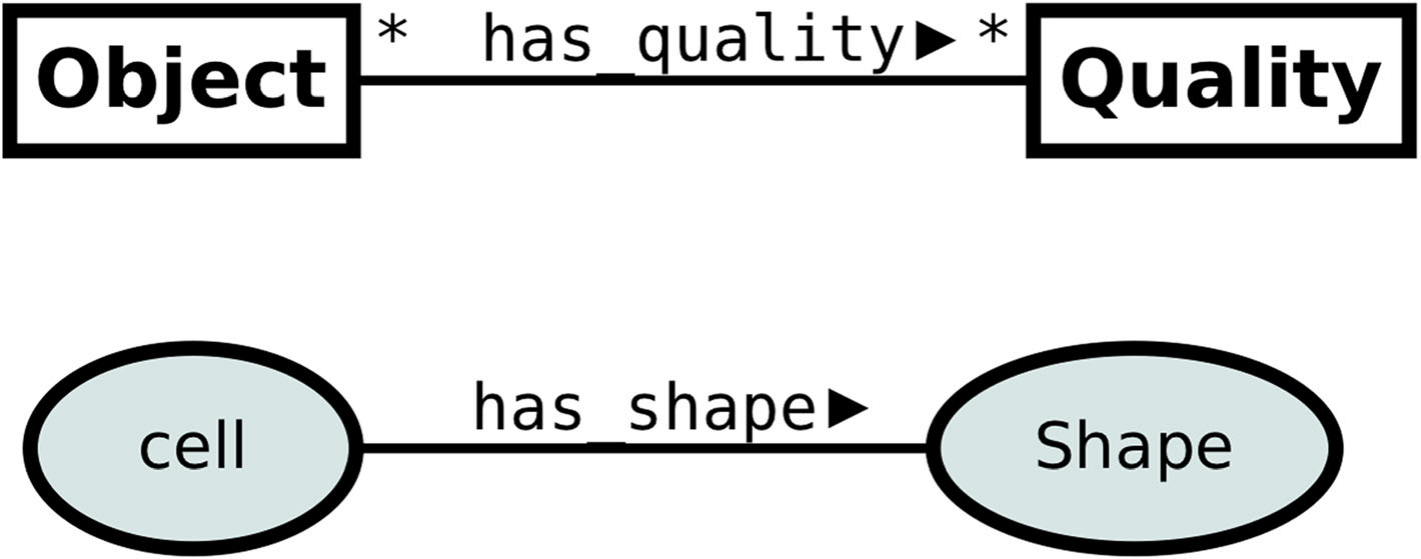
Pattern 1: Quality assignment modeled as OWL property. The upper part of the figure presents a semi-UML diagram depicting the categories used in the pattern. The bottom part presents the application of the pattern to our domain of interest

Figure 3 presents a straightforward approach to modeling qualities in OWL: objects are modeled as OWL Classes, Qualities as OWL Classes or Datatypes and Quality Ascriptions - as Object Properties or Datatype Properties, respectively. The upper part of Fig. 3 presents a UML diagram depicting the pattern itself. The application of the pattern to our use-case is shown in the bottom part of Fig. 3. For instance, a shape of a cell is modeled by owl:ObjectProperty named has_shape, linking an owl:Class Cell with an owl:Class Shape.^2^

Utilizing this pattern, an individual cell and its shape can be defined in turtle notation [49] as follows:

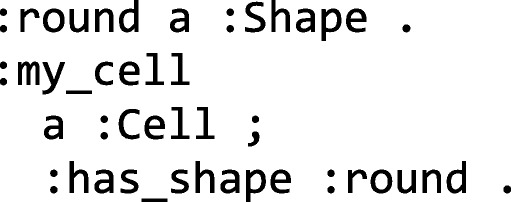



The major advantage of pattern 1 is its simplicity, the limited number of entities and the ease of extension. Unfortunately, this pattern does not allow for representing the change of qualities over time, e.g. the change of a cell’s shape from round to elongated.

**Fig. 4:**
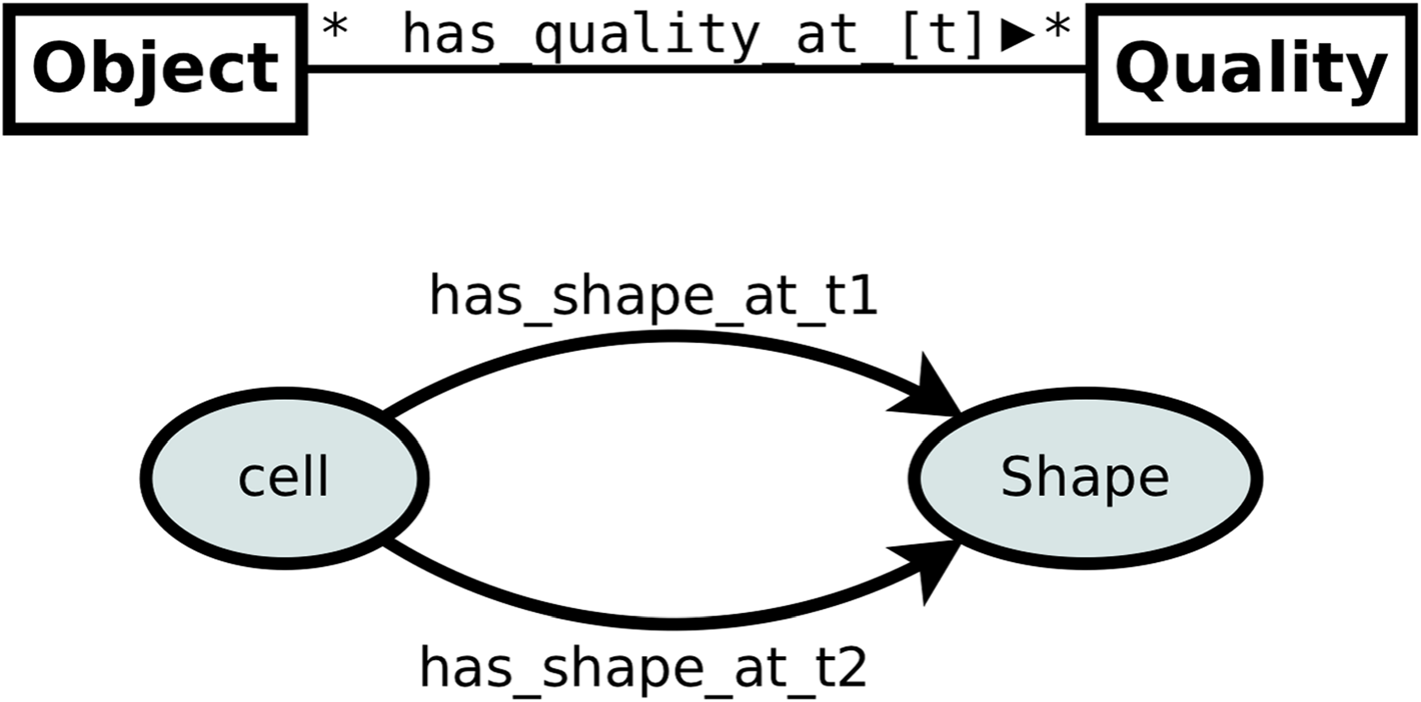
Pattern 2: Quality assignment modeled as time-indexed OWL property

In order to overcome the limitations of pattern 1 and to model the change of an object’s qualities over time one can extend pattern 1 by adding a temporal index to the Quality Assignment property as presented on Fig. 4. For instance, a class *Cell* linked with a class *Shape* by means of two distinct OWL properties: *has_shape_at_t1* and *has_shape_at_t2*, denotes that a cell has a different shape at time *t*_1_ and *t*_2_, respectively.

With the help of that pattern one can easily model the change of cell shape:

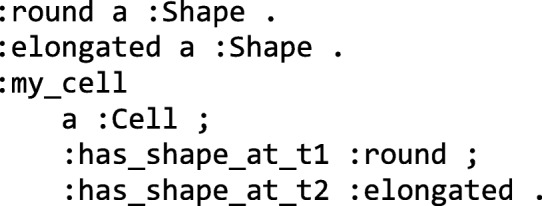



This approach is simple and works well in situations where the number of time indexes is limited or when there is some idiosyncratic time index, as for instance the G2 checkpoint and Meta-phase checkpoint in the cell cycle. Then, the change of shape can be modeled simply by means of two distinct OWL properties: *has_quality_at_G2_checkpoint* and *has_quality_at_Metaphase_checkpoint*. Unfortunately, this pattern is not applicable to our use case, since in cell tracking experiments the number of observations can be very large for a single experiment. Additionally, time indices are not known a priori. Therefore, the application of pattern 2 would require the adjustment of the T-box for each experiment. Moreover, it would result in hundreds of quality assignment properties, which is hardly maintainable.

## Methods

To find an optimal pattern for representing change in biomedical ontologies encoded in OWL we base our analysis on three common criteria for software systems, i.e. scalability, extensibility and adequacy. The first is a common criterion for benchmarking system performance with respect to a growing amount of work. The question to be posed in the context of an ontology is: How does it scale to accommodate an increasing amount of data and, in particular, is the number of entities kept to the minimum even in situations when the amount of information increases? This corresponds to Ockham’s razor [50], a principle broadly adopted to data modelling according to which the number of entities must not be multiplied beyond necessity. That principle entails two rules of database design, i.e. avoidance of data redundancy and simplicity [26].

Extensibility is the second measurement of software architecture anticipating the future growth of the software. In the context of ontology engineering it can be judged by analysing if and how new information can be incorporated into the ontology, without or with a minimal need of reorganizing the existing knowledge base.

Adequacy is a well know criterion of data modelling, also called *faithfulness* [26], which boils down to the rule that model elements should reflect reality. The principle can be verified by examining the following questions: How far do the constructs of the ontology reflect the elements of the domain and is the ontology comprehensible to domain experts (who are often non-technicians)? Although the last criterion is subjective in nature, it works well in practice, especially in situations where ontology constructs reflect *tangible* elements of the domain. It should be noted that the criterion of adequacy in the current paper is understood and applied in purely engineering terms; we do not aim to contribute to the philosophically-oriented discussion on the nature of the elements of the modeled domain, i.e. on realism vs. idealism vs. conceptualism [51, 52].

## Results

### Patterns for modeling qualities

In the current section we review two patterns frequently proposed for modeling temporal information, i.e. reification of n-ary relations and 4d fluents. First, we present the patterns and then discuss their application in three scenarios of distinct temporal distribution of qualities.

#### Reification

The reification of n-ary relations is a popular strategy for modeling temporally changing information. It interprets a time-indexed quality as a 3-ary relationship linking an object, its quality and the time at which the quality is assigned to the object. Next, the relation is reified and introduced to the model as a class.

**Fig. 5:**
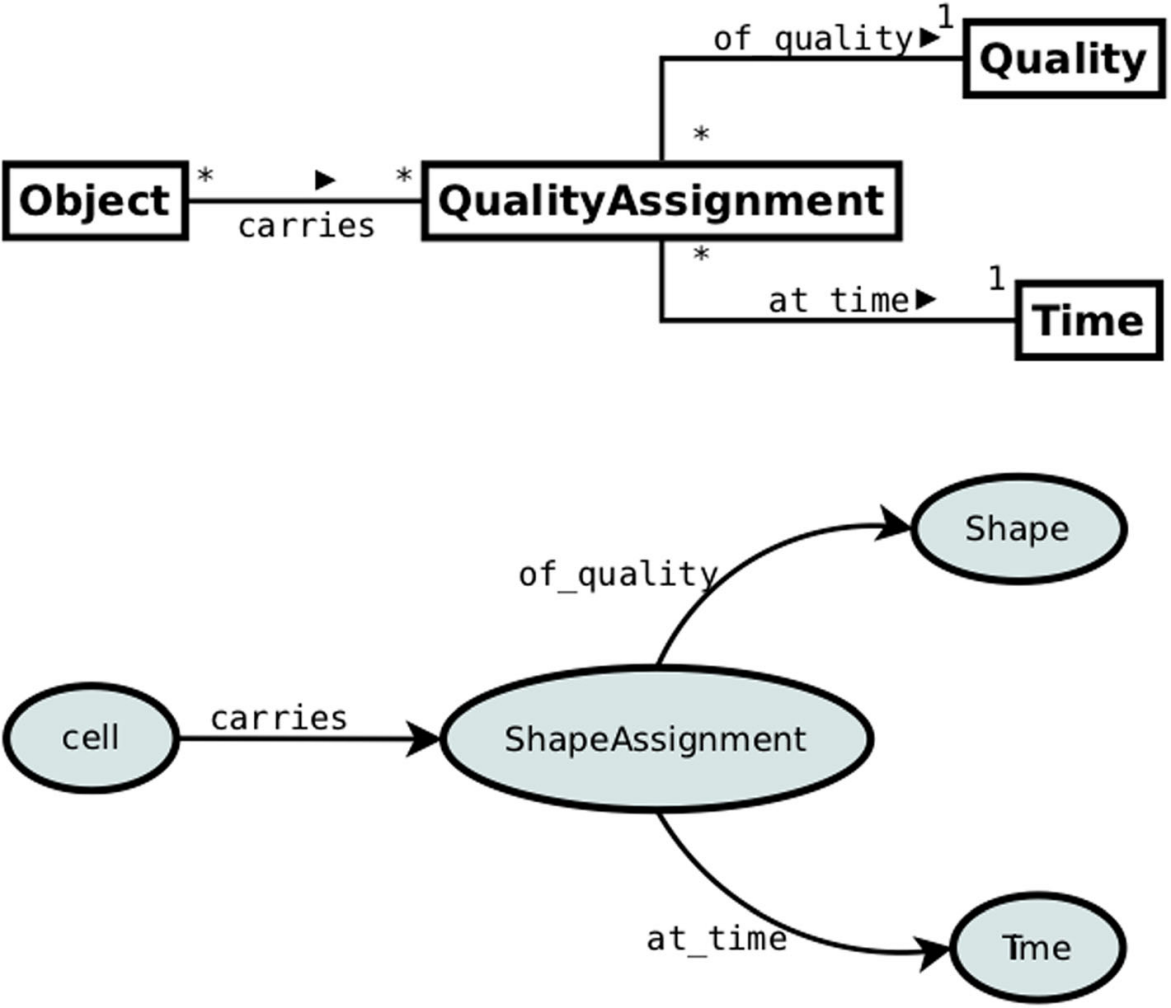
Pattern 3: Quality assignment modeled as time-indexed OWL class

Pattern 3 depicted in Fig. 5 presents the application of this reification strategy to our use case. In contrast to patterns 1 and 2, a Quality Assignment is not modeled as owl:ObjectProperty but instead as a reified owl:Class acting as a proxy between an Object and its Quality.

A reified QA represents a specific assignment of a Quality to a particular Object and as such is dependent on both the Object and the Quality. That means that each Quality Assignment is inherent in exactly *one* Object and is the assignment of exactly *one* Quality. The former constraint is represented by the cardinality restriction on the *carries* link between Object and Quality Assignment and the latter - by the cardinality restriction on the *of_quality* link between Quality Assignment and Quality. Time-index is attributed directly to QA by means of the *at_time* property linking QA with the *Time* class.

The bottom part of Fig. 5 illustrates the application of pattern 3 to the CTO use-case. The object property *carries* links the *Cell* class with the *ShapeAssignment* class, which is a subclass of Quality Assignment. *ShapeAssignment* represents a quality assignment at a given time and has two OWL properties: *of_quality* and *at_time*. The former specifies the value of a quality, i.e. a specific shape, whereas the latter - the time index of the parameter.^3^

This pattern can be applied to annotate a single cell with two distinct shapes at two different time points:

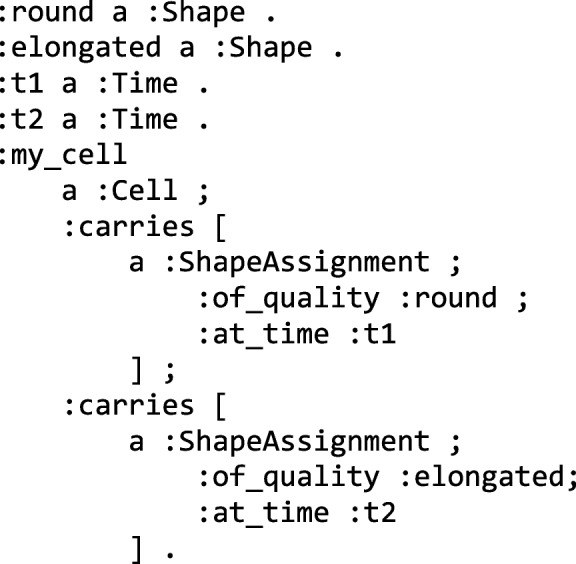



**Fig. 6:**
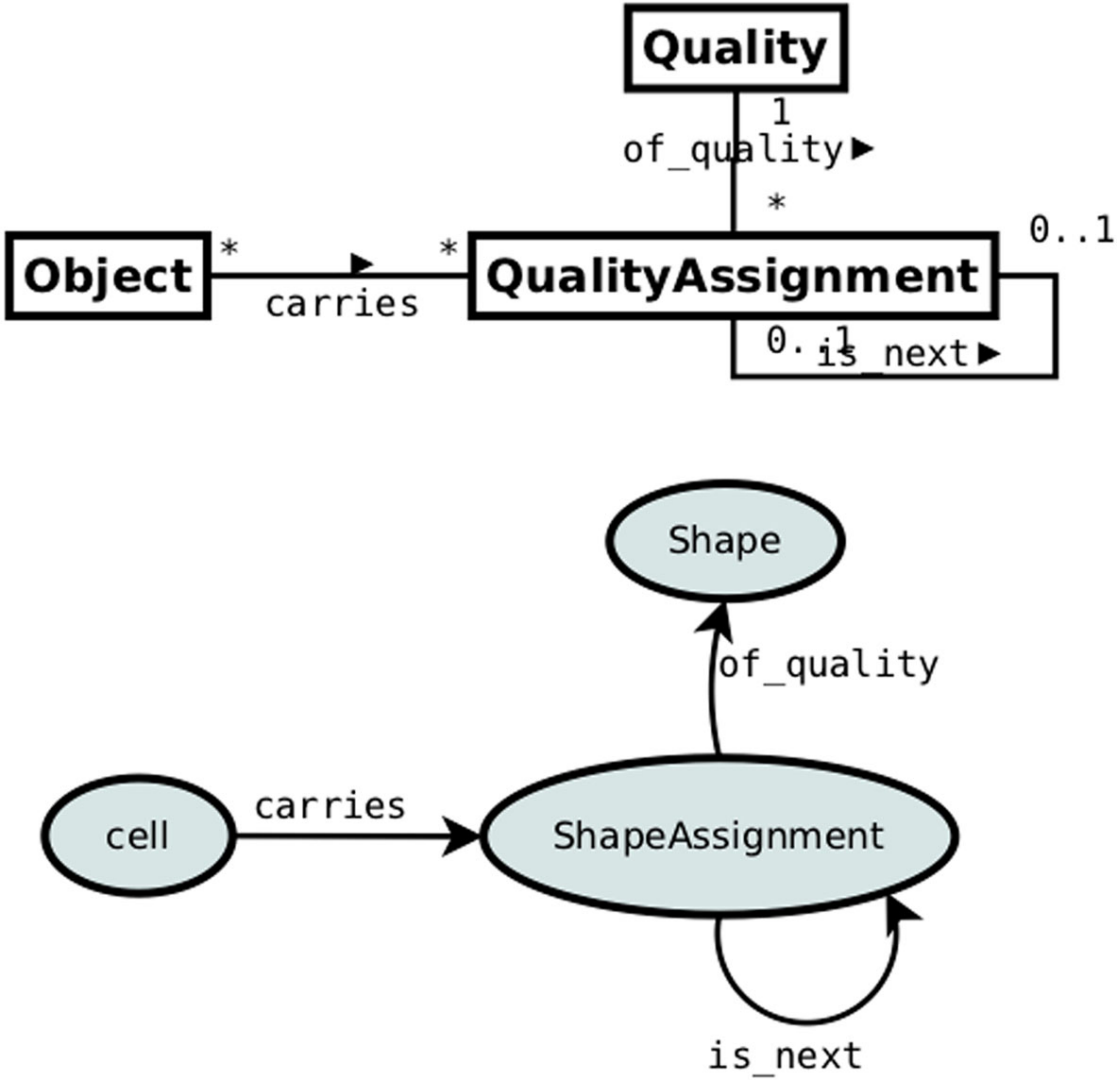
Pattern 4: Temporally ordered quality assignments

In many situations it is not the time index of quality assignments that is relevant but only their t8emporal order. This may also be true for some cell tracking experiments. In such cases pattern 3 can be simplified: in the upper part of Fig. 6 the property *at_time* and the class *Time* can be replaced with the property *is_next*, establishing the temporal order of quality assignments. The implementation of this pattern to our case is presented in the bottom part of Fig. 6.

Pattern 3 overcomes the limitations of patterns 1 and 2 reported above, since time-indexed quality value assignments are represented as instances only. Thus, even in situations where many time-indexed value assignments occur, the number of classes and properties in the ontology remains constant (and is relatively low).

On the other hand, as observed in [31], the model introduces additional OWL classes and OWL properties for representing time-indexed quality ascriptions, reducing its lucidity.

#### 4d Fluents

**Fig. 7:**
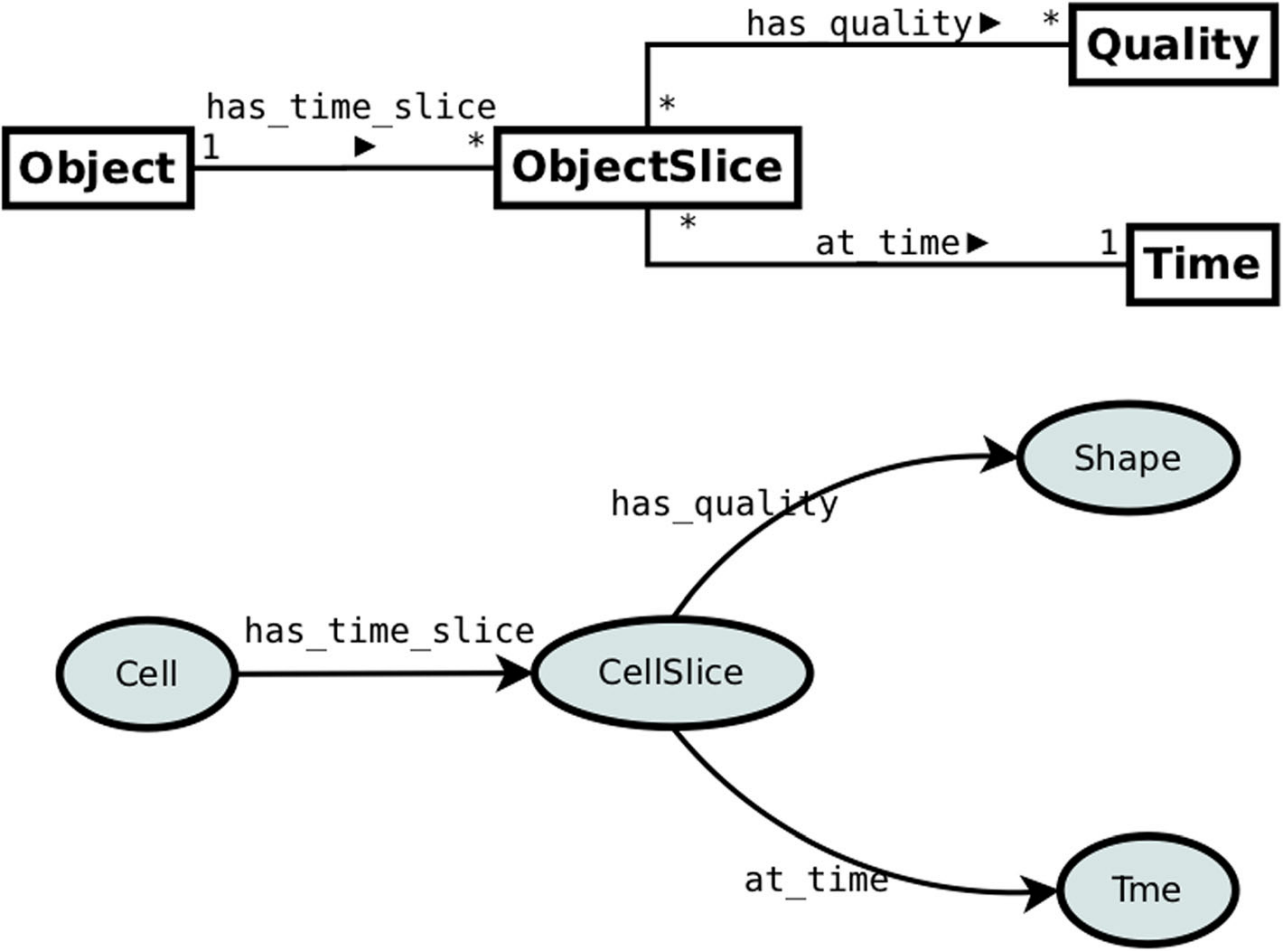
Pattern 5: Reified 4D fluents

An alternative to the n-ary relation reification is the so-called 4d fluents pattern [30]. It is inspired by four-dimensionalism [29], a philosophical theory explaining the persistence of objects through time, called perdurance, in analogy to their extension in space: similarly to an object occupying some space *s* having parts occupying parts of *s*, an object occupying some time *t* may have temporal parts occupying parts of *t*. In that understanding, time-extended objects are considered as the so-called *4d worms*, which can be sliced into temporal parts, as 3d objects can be sliced into their spatial parts. The top-part of Fig. 7 presents a 4d pattern. In contrast to the reification pattern, the idea behind 4d fluents is not to reify a temporally indexed relation but instead a temporal part of an object. For instance, in order to model the fact that a cell *c* has a round shape at time *t*_1_ one can reify a temporal part of *c* and then assign a quality directly to the reified part:

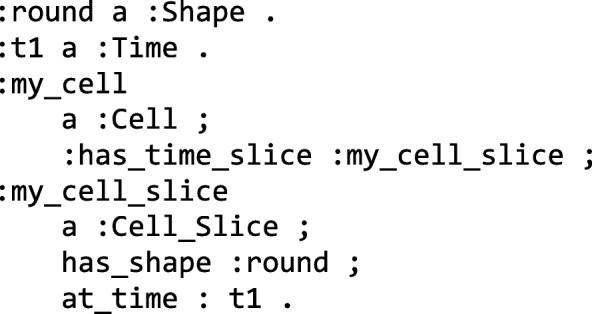



Conceptually, the two patterns seem quite distinct, yet when comparing Fig. 5 and Fig. 7 one can observe that structurally they are almost the same and both are based on introducing an association class. The only structural difference is the cardinality constraint determining the number of qualities linked to the reified class. In the reification pattern it is 1, whereas in the 4d fluents pattern it is 0..n. Therefore, when modeling an object’s single quality assignment, both patterns are in fact equal.

The difference between the patterns can be well illustrated when applying the patterns to relations rather than quality assignments. Let us consider the CTO relation of cell-cell contact, denoting the fact that two cells touch each other. In order to model a temporally indexed cell-cell contact, the reification pattern requires the introduction of a single reified time-indexed relation, whereas the fluent pattern would introduce a reified and time-indexed slice for each cell participating in the contact.

The difference between the patterns can also be observed in cases where numerous quality assignments are being represented, which is in fact the real challenge of ontology engineering. Therefore, we will analyze the patterns using three different cases of temporal distribution of qualities:

- Temporally non-overlapping quality assignments. For instance, a cell can have an oval shape at one time and an elongated shape at another, but it can never have both shapes at the same time.
- Temporally equal quality assignments. Thit is a typical scenario in time lapse experiments where at a single time point numerous distinct qualities are observed, e.g. shape, location, etc.
- Temporally overlapping, but not temporally equal quality assignments. This is a common situation when qualities change independently from one another, as is the case with the location and shape of a cell.

### Temporally non-overlapping quality assignments of a single quality

As a starting point, we consider the simplest case, in which no two quality assignments of an object are located at the same temporal location. Such a situation is natural for many qualities when considered separately, e.g. typically a cell has a single location or a single shape at any given time. This scenario is often assumed in the works devoted to the modeling of temporal information, e.g. in [24, 30, 31]. In such a case it can be easily observed that both patterns behave the same, in fact there is no difference when applying them. Modeling *n* quality assignments of a single quality of a single object we need to introduce *n* instances of a reified class in both cases.

Both models are equally extensible, i.e. to introduce a new characteristic a new instance must be added to the model. Finally, the adequacy of both solutions seems to be merely a matter of personal taste since the choice between the patterns generates no structural differences in the models.

### Temporally equal quality assignments

The above discussion is justified when considering a single quality in isolation. Yet, when considering numerous qualities of an object, it is clear that there can be two or more quality assignments which overlap temporally, e.g. a cell at a given time point can have some location and some shape. This is a typical scenario in cell tracking experiments, where at a single time point more than one quality is observed. In such cases the application of the reification pattern results in a model with redundantly time-indexed Quality Assignments: for each quality observed at a given time point a separate Quality Assignment instance has to be introduced.

It seems that the 4d fluents pattern solves that problem. Time slices in their simplest form are temporal parts of objects having an arbitrary temporal extension (usually considered an interval). An alternative approach, present e.g. in the General Formal Ontology (GFO), introduces temporal particles located at discrete time points (the so-called presentials) which are distinct from time extended slices [45]. In GFO, a presential is an entity that is wholly present at a single time point. For instance, a cell observed at a single time point would be considered a presential cell. A presential may have multiple assigned qualities, all present at the same time point as the presential which carries them. Thus, a presential is a snapshot of a time extended entity, i.e. a cell observed at a single time point can be considered a snapshot of a time extended cell.

**Fig. 8:**
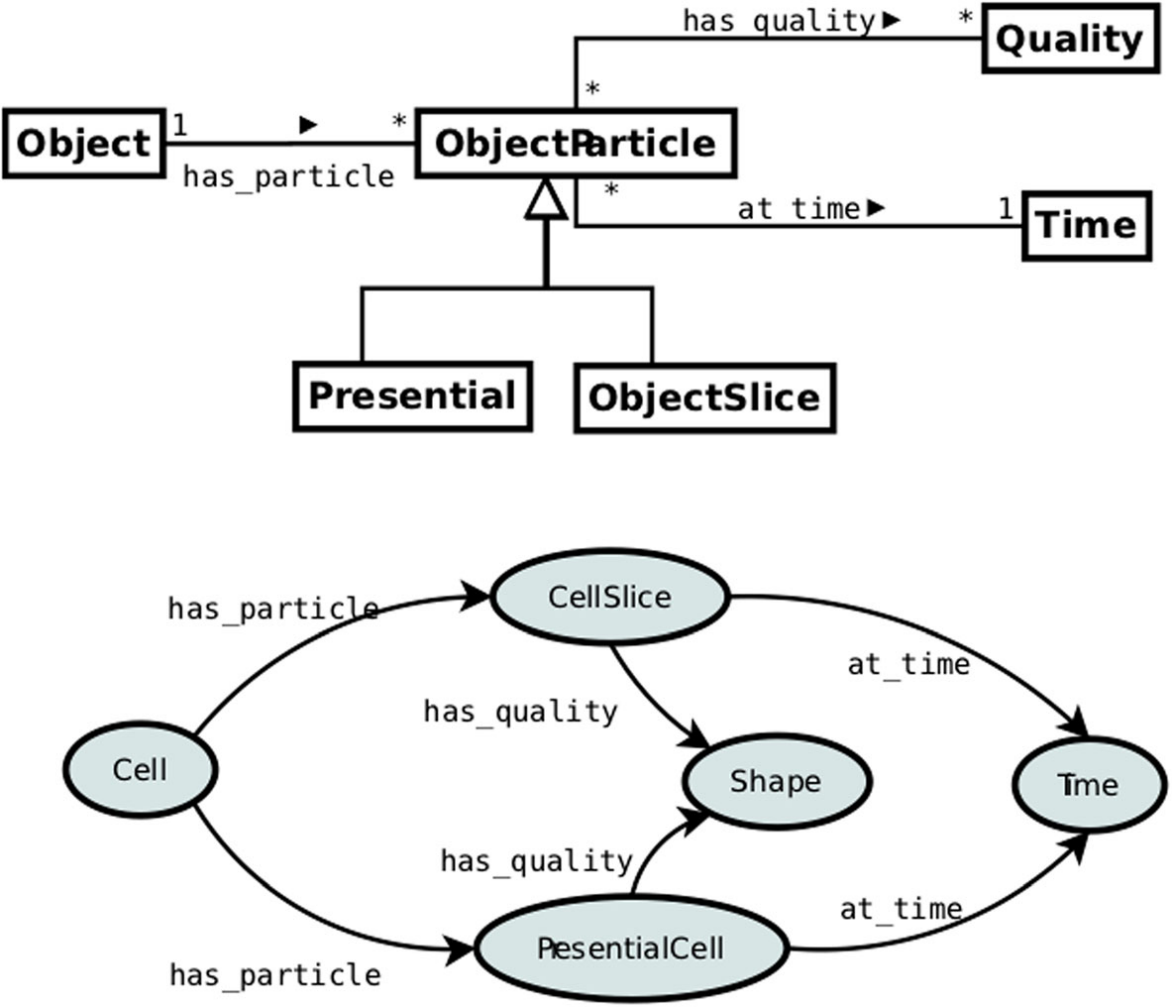
Pattern 6: Generalized 4D fluents. Presentials and Slices

Figure 8 presents the pattern for modeling time slices and presentials where both are considered temporal particles of objects. Based on that pattern a modeler can utilize both time interval slices and/or presentials, depending on the actual needs.

The annotation of an individual cell using the presential pattern would look as presented below:

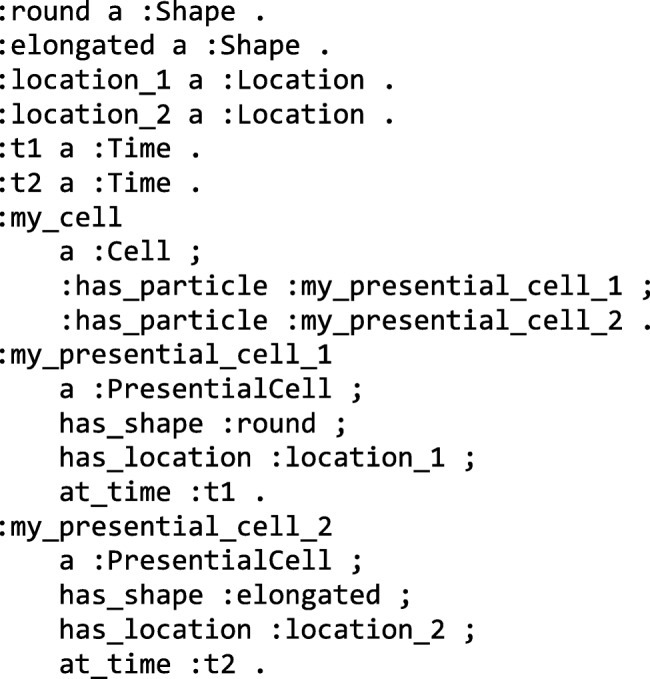



In contrast to the reification pattern, the presential pattern reduces the number of instances introduced to the model. Instead of reifying each quality assignment at a given time point, all coinciding quality assignments are modeled with the help of a single presential instance.

4d fluents also scales better: each new quality assignment added to the reification-based model requires a new instance, whereas in the case of the 4d-based model no additional instance is needed when a time slice with the same time index already exists in the model.

The frugality of the 4d pattern seems to make it more intuitive than the reification pattern, especially in the case of cell tracking experiments where the presential cells are the entities physically represented in the acquired images. Thus, they can be easily identified and it seems quite natural to reify them.

### Temporally overlapping characteristics

In cell tracking experiments the qualities of enduring cells are deduced on the basis of a sequence of observed presential cells and their qualities. For instance, if a cell is observed to have a round shape over the sequence taken at time points *t*_1_, *t*_2_,.., *t*_*n*_, then typically one can deduce that the cell has a round shape during the whole time interval (*t*_1_, *t*_*n*_). Clearly, in the case of numerous mutually independent quality assignments it may turn out that the temporal extensions of many of them may overlap. For instance, during a time interval (*t*_1_, *t*_5_) a cell may remain in location *l*_1_ but its shape may change from round in (*t*_1_, *t*_3_) to elongated in (*t*_3_, *t*_5_). That results in two shape quality assignments overlapping with the location quality assignment. This situation is clearly visible when the reification pattern is used, as for each observed quality assignment a reified instance is introduced.

However, when turning to 4d fluents, the first observation we make is that the adaptation of the pattern to this case is not as straightforward as in previous cases, when it was relatively easy to say what the cell’s time slice is, namely, a presential cell observable in an image and thus having its own identity. However, in the current case we want to reify not the presential (observable) cells but instead the time extended, temporal parts of cells. This raises the following question: What is a temporal part of a cell and what rules drive the slicing of an object (a cell) into its temporal parts?

It seems that at least two strategies for introducing temporal parts could come in handy. The first is based on the principal idea of 4-dimensionalism, namely that a time extended entity can be sliced into temporal slices in such a way that each slice fully represents the sliced object at a given time. This means that all qualities assigned to an object within the time span of a slice are attributed to the slice directly. We call this type of slicing *vertical*.

Unfortunately, modeling temporally overlapping quality assignments with vertical slices easily leads to serious redundancy and is hardly maintainable. This is due to the fact that slices are overlapping and each one represents an object in full at a given time and as such it carries all qualities attributed to the object during the slice’s lifetime.

An intuitive solution to fix this problem would be to prohibit the overlapping of slices. This results in a model in which a time extended entity is sliced into non-overlapping slices so that the sum of all the parts constitutes the full lifespan of the object.

Let us illustrate this strategy with an example, starting with the model of a cell remaining in location *l*_1_ during the interval (*t*_1_, *t*_5_):

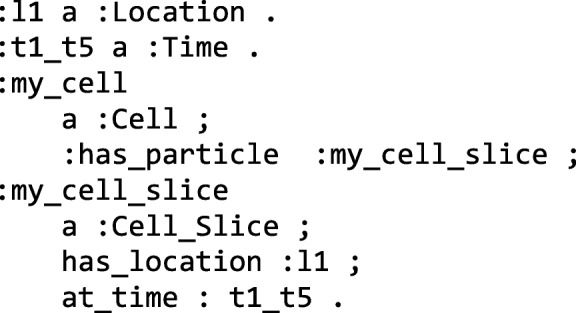



Now, let us assume that we add to our model a new observation (fact) that the cell changes its shape from round in (*t*_1_, *t*_3_) to elongated in (*t*_3_, *t*_5_). If we add that observation to our model, we end up with two additional time particles depicting the location of the cell: one ending at *t*_3_ and the other starting at *t*_3_, thus both new particles are overlapping with:my_cell_slice. In order to fix this, one could reorganize the time slices into two non-overlapping slices, the first representing the state of the cell being round and located in *l*_1_ and the second - the state of the cell being elongated and located in *l*_2_.

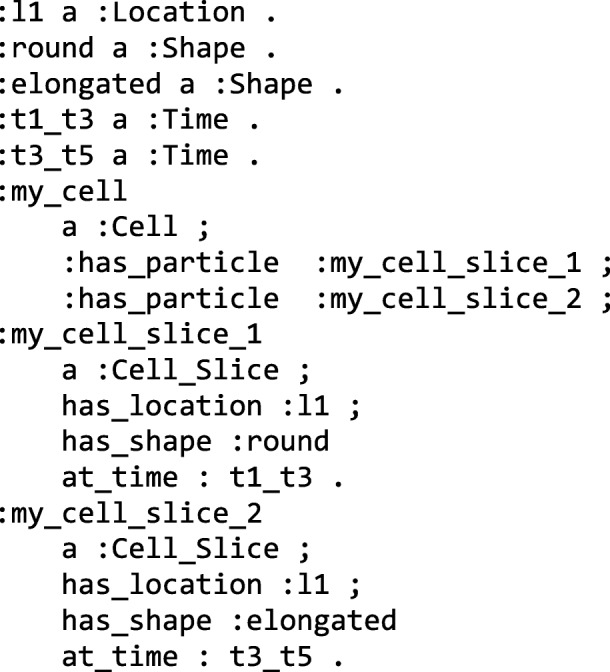



Unfortunately, this strategy has its problems. As it can be seen from the above example, the change of any of the qualities may entail the reorganization of the object’s slices. That leads to a proliferation of slices, but more importantly, it makes the knowledge base strongly coupled and thus harder to extend, i.e. the addition of new information entails the reorganization of previous knowledge.

In addition, despite the fact that the strategy solves the problem of the model’s redundancy, it still results in heavily overloaded models. As presented on the listing above, each slice carries a full specification of the cell at a given time, even for those qualities which remain constant across many slices.

In order to overcome those limitations an alternative interpretation of 4d fluents could be considered: an entity could be sliced not only vertically, i.e. along the time dimension, but also horizontally, i.e. along its quality assignments. That way a slice does not fully represent an object at a given time but only some of its aspects, e.g. that a cell is located at *l*_1_ at *t*_1_ - *t*_2_. Thus, a slice is a kind of temporally indexed reified attribute of an entity.

That interpretation fixes the problem of model redundancy, but it also blurs the difference between the 4d fluents and the reification pattern, since a time slice now represents some quality assignment, i.e. some temporally indexed attribute of an entity. The actual difference between such an interpretation of time slices and reified quality assignments is hidden in the cardinality constraint on the quality role (presented in Fig. 5 and Fig. 7). While the reified quality assignment links an object with a *single* quality, a slice can link an object with *multiple* qualities when quality assignments overlap temporally. Thus, if we add the fact of a cell’s size, which is temporally equal to that of its shape, we are not forced to introduce a new temporal particle but it is sufficient to add that fact to: my_cell_slice_2:

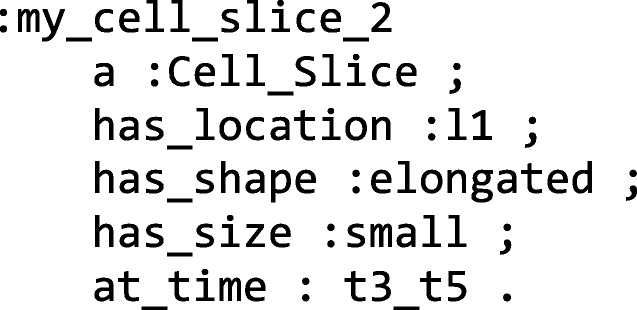



## Discussion

**Fig. 9:**
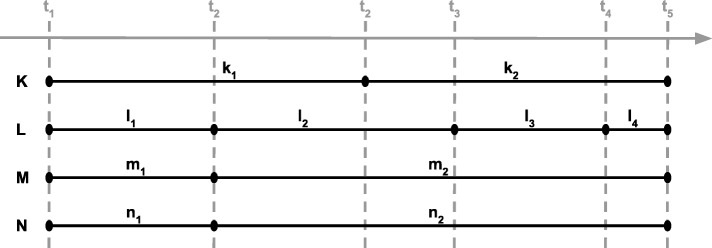
A fragment of simulated cell tracking experiment results presenting changes of qualities K, L, M, N over time t_1–5_

Our analysis shows that there is no single best choice with respect to simplicity, scalability, extensibility and adequateness for modeling a change of qualities over time. Table 1 provides a condensed summary of the discussed patterns and their flavours. Additionally, as the ontology of cell tracking experiments is still under development and relevant amounts of annotated data are currently lacking, we provide a synthetic example in Table 2 as a benchmark for the performance of discussed patterns. We simulated data of a single cell undergoing a parallel changes of four qualities K, L, M, N over time t_1–5_. Since the size of A-box depends on the distribution of quality changes we have simulated several schemas of change as depicted on Fig. 9, i.e. qualities K and L changes independently from all others, from values k_1_ to k_2_ and from l_1_ throughout l_2_, l_3_, l_4_ to l_5_, respectively. M and N, in turn, undergo a change simultaneously.

**Table 1:** Overview of patterns and their performance in representation of change of qualities

Pattern	Overview	T-box Simplicity (wrt number of t-box elements)	A-box simplicity (wrt number of A-box elements)	Extensibility/maintainability	Adequateness
OWL property	A default OWL handling of qualities.	*Very High* - no additional classes or properties.	*Very High -* no reified instances.	*High*	*Very low* - no support for representing change of quality assignments.
T-indexed OWL property	The pattern is simple and works well for limited number of time indexes or for idiosyncratic time index.	*Low* for cell tracking experiments - the number of object properties is high and proportional to the number of t-indexes	*Low* for cell tracking due to high number of t-indexed property axioms.	*Low* for cell tracking - requires the adjustment of the T-box for each experiment. Complexity of T-box implies poor maintainability.	*High* - no reified modeling artifact must be introduced..
Reification	The pattern overcomes the limitations of patterns above - time-indexed quality value assignments are represented as reified instances only. Therefore, even in situations where many time-indexed value assignments occur, the size of T-box is constant.	*Moderate* - the number of reified quality value assignment classes equals the number of qualities.	*Moderate* - the number of reified instances equals the product of objects, qualities and t-indexes. For temporally equal quality assignments it causes redundancy, i.e. for each quality observed at a given time point a separate Quality Assignment instance has to be introduced.	*Moderate* - a new quality assignment requires a new instance of a reified class.	*Moderate* - reified quality assignments do not correspond to any tangible objects in a domain of cell tracking but are mere modeling constructs.
Reification w/ temporally ordered assignments	The pattern simplifies and restricts the expressivity of the reification pattern by no explicit representation of time entities but only a temporal order of quality value assignments.	*High* - same as above wrt quality assignment. Additionally, no classes for time entities are introduced.	Same as above wrt quality assignment. Additionally, no instances for time entities are introduced.	*Moderate* - same as above.	*Moderate* - same as above.
4d fluents (vertical) / states	The pattern, in contrast to the reification pattern, reifies not a temporally indexed relation but instead a temporal part of an object which can be interpreted as a state of an object.	*High* - number of reified classes is reduced to the number of object types	*Very High* for temporally equal qualities- instead of reifying each quality assignment at a given time, all coinciding quality assignments are represented as a a single instance. *Moderate* for temporally overlapping qualities - the number of reified quality instances is a function of quality assignment changes and depends on the distribution of changes.	*Very High* for temporally equal qualities - no additional instance is needed when a time slice with the same time index already exists in the model. *Low* for temporally overlapping qualities - new quality assignment results in proliferation and reorganization of reified instances (slices) and multiplication of property axioms for non-changing qualities.	*High* for cell tracking experiments - presential cells represent the domain adequately - they are the cells represented in the acquired images or the temporally indexed states of cells.
4d fluents (horizontal)	The pattern, overcomes the limitations of the vertical 4d fluents pattern in representing the temporally overlapping qualities by the introduction of horizontal slicing such that a reified entity represents some quality assignment.	*High* - same as above.	*Very High* for temporally equal qualities - same as above. *High* for temporally overlapping qualities - in contrast to the reification pattern, it enables the bundling of temporally equal characteristics into a single entity, which limits the number of reified entities	*Very High* for temporally equal qualities - same as above. *High*- for cell overlapping qualities solves the problem of the vertical 4d pattern. Additionally, in contrast to the reification pattern a new quality assignment requires a new instance only if a time slice for a given time does not exist yet in A-box.	*Low* - time slices do not reflect cells observed on time points but instead collections of temporally equal quality assignments. A non-obvious interpretation of the association entity limits the intuitiveness of the model.

**Table 2:** Amount of elements for a fragment of ontology representing the change of qualities of a single cell illustrated in Fig. 9

	Classes	Object properties	Individuals	Object Property Expressions
T-indexed OWL property	5	7	11	7
Reification Pattern	10 (7)	3	28	30
4D Vertical	7	3	21	30
4D Horizontal	7	3	25	24

Below we elaborate in detail on two of the presented patterns, which are our main focus in the current paper, i.e. the reification and the 4d fluents patterns. These patterns perform differently depending on the temporal distribution of quality assignments. However, both patterns result in the same model in the case where quality assignments are not temporally overlapping. This situation seems, however, merely theoretical and in real life cases it is to be expected that numerous qualities have to be modeled (as it is also the case in the cell tracking ontology). In fact, the introduction of numerous qualities which are temporally overlapping or equal is the major source of modelling complexity.

In cases where quality assignments are temporally equal, the 4d fluents pattern performs better. Firstly, the expected size of T-box is smaller in case of the 4d fluents pattern as the number of reified classes is equal to the number of domain classes/types having the qualities attributed, whereas, in case of the reification pattern, it is equal to the number of qualities, which, in turn is typically much higher than the number of classes/types.^4^ In fact, in case of the cell tracking ontology the number of classes is reduced to one as we are only interested in cells. The size of A-box is also expected smaller for the 4d fluents pattern as the number of reified instances is the product of objects and t-indexes and the number of qualities has no additional influence in contrast to the reification pattern. The extension and maintainability of such models is also simpler than of models based on the reification pattern, since adding new quality assignments requires no additional (reified) model element. Especially in cases where temporal slices are tangible objects (as in cell tracking experiments), the number of reified entities is lower and the reified presentials are well-grounded domain concepts, which in turn increases the adequateness of the model.

In cases of temporally overlapping quality assignments the application of 4d fluents is not straightforward. It could come in variants (a) vertical 4d fluents: a fully specified non-overlapping slices, and (b) horizontal 4d fluents: a not fully specified overlapping slices. For both variants of the pattern the size of T-box is expected to be smaller compared to the reification pattern. The size of the A-box varies depending on the distribution of the quality changes over time, yet the total sum of instances and property axioms is higher for both variants than for the reification pattern.

Here, the first variant does not work well since it is hardly extensible, i.e. adding a quality assignment which is temporally overlapping with existing ones requires the reorganization of the A-box - it results in proliferation and reorganization of reified instances (slices) and multiplication of property axioms for non-changing qualities. For instance, the attribution of m_2_ over t_2–5_ must be split into three axioms, one for each vertical slice.

The second variant solves that problem, as there is no need of redundant quality assignments for non changing qualities. Additionally, in contrast to the reification pattern, it enables the bundling of temporally equal characteristics into a single entity, which limits the number of reified entities. New quality assignment only requires introduction of a new instance if a time slice for a given time is not yet present. However, a drawback of such an approach is the non-obvious interpretation of the association entity, which limits the intuitiveness and adequacy of the model.

From the above considerations, we see that a presential variant of the 4d fluents pattern is naturally applicable to the case of temporally equal quality assignments, especially in time lapse experiments where the presential cells are tangible/observable objects. Yet, in cases of non-equal but overlapping quality assignments, the application of 4d fluents is not straightforward, demonstrating the major weakness of a 4d approach: it is not common-sense. In [30] the authors observe that it is not very natural to convert statements such as “Joe walked into the room” into their 4d equivalents such as “A temporal part of Joe walked into a temporal part of the room”. It seems that four dimensionalism has several weaknesses, also on the level of philosophical theory underlying the 4d fluents pattern, and the discussion is still not settled [53]. One of the open problems is the identity of four-dimensional entities. For a four-dimensionalist, the identity of an entity resides in its unchanging temporal parts, but, on the other hand, one can argue that an entity has different temporal parts at different times. In contrast, three-dimensionalism seems more common-sense in that respect. 3d objects are considered to be identical over time, and only their properties change over time with no harm to the criteria constituting the identity of 3d objects.

Therefore, one may still ask if it is possible to get the benefits of the 4d-pattern without slipping into the 4-d interpretation of time extended entities. We believe that some well known approaches in the area of software engineering permit a modeler to abandon a 4d account of reality, sticking to the 3d approach and still obtaining a similar output as the 4d pattern.

For instance, the second variant of the 4d pattern can be successfully represented using *state modeling*, a technique originating from finite-state machines, which, due to its intuitiveness, is applied far beyond hardware and software engineering. In state modeling the behaviour of an object (a system) is modeled with the help of the states the object can be in. An object’s state corresponds to a phase of the execution of the state machine during which some invariable condition holds. A state can be defined by the attributes of the object and their respective values. For instance, a state of a cell can be defined by the cell’s shape and location.

The change of an object’s state is modeled with the help of a transition, which is a directed arc linking the source state with the target state. An object can change its state over time but at any given time an object can only be in a single state. That corresponds to the second variant of the 4d pattern, where time slices are non-overlapping.

Thus, a change of quality assignments over time can be interpreted in terms of a change in the object’s state. In that sense, a model presenting temporal non-overlapping slices of a cell can be easily reformulated with the help of state modeling:

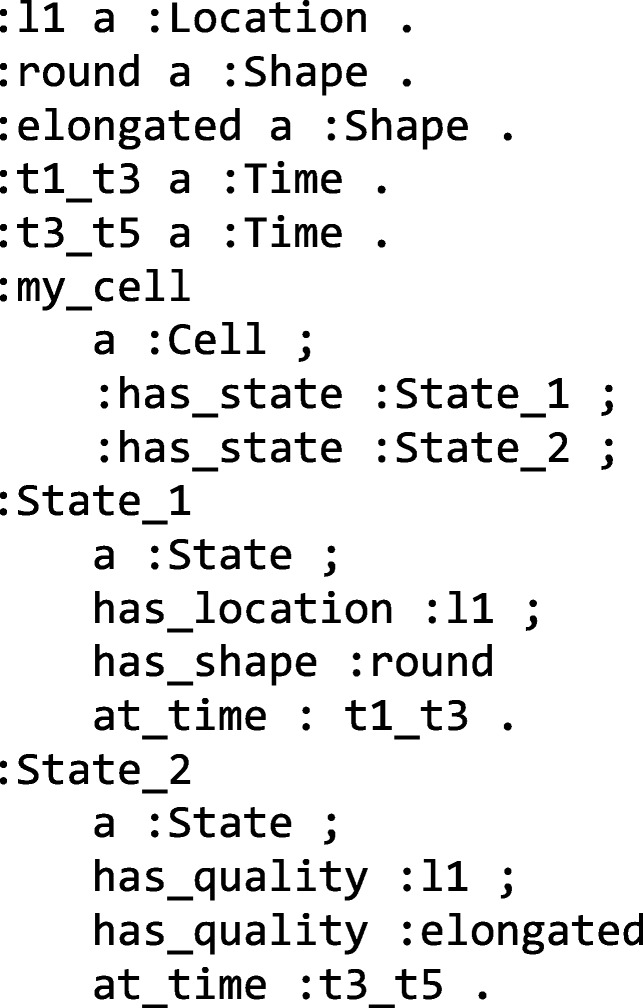



In contrast to the previous model, the new model does not use the notion of time slices, which requires a 4-d interpretation of entities. Instead, a collection of quality assignments constitutes the state of the object.

Another alternative approach is the actor-role pattern, also known as actor-participant pattern [54]. The pattern originates from software engineering, but can be applied in the context of ontology engineering as well [55]. In principle, the pattern is used to decouple the identity (the actor) from the behavior (the role). The pattern consists of two entities, an actor and a role, linked by a one-to-many relationship. An actor is an entity which has an identity and attributed non-changing characteristics. A role is existentially dependent on an actor and bundles all characteristics attributed to the actor in the context in which he plays a role. An actor can have many roles both simultaneously as well as sequentially, whereas a role is always a role of a single actor. A classical example of an actor-role pattern are social roles such as e.g. a person (an actor) having different roles such as a student, a driver or an employee. A role pattern has been extended by some authors to an actor-role-context pattern, introducing an additional entity representing a context in which an actor plays a given role [55, 56].

The modeling of temporally indexed quality assignments with the role pattern results in a model analogous to the third variant of the 4d pattern. In that sense, horizontal and vertical slices of an object can be interpreted as roles of the object in the context of a bundle of qualities. Hence, each bundle of temporally equal quality assignments is represented as a separate temporally indexed role. Thus, each role represents some aspect of an entity at a given time, but, in contrast to the state modeling pattern, roles can temporally overlap and a single role does not provide a full specification of the object’s characteristics at a given time, but only those relevant in its context.

Summing up, the 4d pattern can be successfully reconstructed with two intuitive and well known patterns, i.e. state and role modeling. This finding can be helpful to modelers not familiar with the philosophical account of 4-dimensionalism or those for whom considering time extended objects in terms of 4d entities could be counter-intuitive.

### Choices adapted to the cell tracking ontology

Our analysis demonstrate that there is no single silver bullet approach to modeling temporally changing information. The key aspects here are the number of time indexes and the actual temporal distribution of the information to be modeled. Based on our analysis we derive the following guidelines for ontology engineers:

- The default handling of OWL properties is most performant but provides no means for modeling changes in quality values and therefore cannot be used when those changes need to be represented.
- The t-indexed property pattern is suited for cases with a limited number of time indices or in case of an idiosyncratic time index.
- The vertical 4d fluents/states pattern is suited for cases with many time indexes and with temporally equal quality assignments. That is a typical setting in domains and applications where states of objects are observed and documented at particular time windows as it is the case of time lapse experiments where cells are imaged at equal time intervals.
- The horizontal 4d fluent and the reification pattern are best suited for the cases with overlapping but not equal quality assignments. This is the case for instance when object qualities change independently one from another, as for instance in situation where the shape of a cell changes independently from its location.

Accordingly, we have developed CTO as a combined approach of n-ary relations and presentials. In CTO, there are two possible types of temporal particles involved: presentials and interval-based particles. The former correspond to tangible objects observable in images and indexed with discrete and, therefore, non-overlapping time points. This is why we have decided to reify them. This reduces the number of entities, since we are not forced to reify each QA on the presential level but instead we can use a straightforward OWL approach to model qualities as properties of presentials. On the other hand, in order to model interval-based, overlapping and dynamically changing quality assignments we have decided to use reified quality assignments.

The combination of both patterns supports at the same time the requirements of Ockham’s razor (the number of entities used for representing presential quality assignments is limited by the usage of presentials) and the extensibility of the ontology (since we use reified time extended quality assignments, there is no need to reorganize the existing ontology after adding new overlapping quality assignments).

In the current paper we focus on a specific use case of modeling the change of an object’s qualities. Yet, we think that the patterns and design choices presented are not restricted to that case: in the development of CTO we have followed the very same principles for modeling the change of relations between cells. One example of such a relation is cell_cell_contact representing adhesion of two or more cells. Here, in analogy to the case of quality assignments (modeling relations indexed with time points), we have decided to follow the presential pattern and we have used the reification pattern to model time-extended relations.

## Conclusions

Biomedical systems are dynamic in their nature; the representation of change is thus one of the fundamental challenges for knowledge engineering in the biomedical domain. The current paper addresses the problem of modeling the change of quality assignments over time in biomedical ontologies encoded in OWL. The paper discusses two patterns for modeling temporally changing information, i.e. n-ry relation reification and 4d fluents. In contrast to the rich literature on the topic, we are not interested in modeling temporally isolated characteristics but an entire web of characteristics in a dynamically changing domain. Concerning an ontology of time, we take a minimal ontological commitment which can be easily fulfilled by various time ontologies, depending on intended granularity of the model. In many cases, OWL-time is sufficient, though there might be situations in which the modeling of a discrete change is needed. In this case, OWL-time is not sufficient and we may use GFO-time (and the corresponding OWL-representation).

We discuss the application of these patterns to the biomedical ontology dedicated to the annotation of cell tracking experiments (which is currently under development). We have analyzed the performance of each solution in three different settings with respect to common criteria of software engineering and data modelling, i.e. scalability, extensibility and adequacy. We have discussed the benefits and drawbacks of each approach as well as the underlying design choices. For each design choice, we have presented possible options and modeling variants.

The lesson learned from this analysis is that there is no single best approach. We demonstrate that the patterns behave differently depending on the temporal distribution of the information modeled. Thus, the optimal model can be obtained by combining the two competitive approaches. The example of CTO demonstrates that both reification and 4d fluents patterns can work hand in hand in a single ontology.

Additionally, we have found that in the (common) case of temporally overlapping quality assignments the application of the 4d fluents pattern can be reconstructed by two alternative patterns well-known in computer science, i.e. the state modeling pattern and the actor-role pattern. This finding can be helpful to those users not familiar with the philosophical discussion on four-dimensionalism to whom considering entities in terms of 4d worms may seem awkward.

Although the discussed patterns are dedicated for OWL, the underlying conceptual choices are generic in nature and we believe that they can be successfully applied to other technologies and formalisms, such as, e.g., UML or ERD. We also expect that the same patterns could be helpful for modeling other types of temporal information, such as temporal relations.

The patterns have been investigated in the context of developing a biomedical ontology dedicated to the annotation of cell tracking experiments. The ontology is intended for integration with software used for annotation of cell tracking results [11, 35–37] (see also Fig. 2). Obviously, there are two main tasks to solve: Firstly, we need a sufficiently expressive annotation ontology (mainly an ontology describing qualities of cellular genealogies), secondly, we need a support for the annotation of time lapse experiments (being sequences of visual frames) by using the concepts of the CTO. The second step could be supported by machine learning methods, though the training data must be provided by experts.

The evolution of ontologies is an important research topic which must be taken into account as existing annotations of time lapse experiments would have to be re-annotated for a new version CTO (1) of the ontology CTO. Here, we would build on the framework for classifying and realizing ontology- versions in the context of ontology evolution as presented in [57].

Finally, modeling of quality value change is not limited to cell tracking experiments, but is a common and non-trivial task across many domains of interest. The analysed patterns are domain-independent and, since a change of quality values is common to many biomedical domains, we believe that the application of these patterns is important in many related problems. In such a setting the patterns discussed can be used for the extension of the existing modeling languages such as e.g. UML and used for the purpose of ontology engineering and conceptual modeling in various applications including modeling of new ontologies as well as refactoring of existing ones. We already realized this approach and proved its utility for a different modeling task of function representation, firstly by introducing the extension into the UML [58] and, secondly, by the application of extended UML for the task of refactoring of the Gene Ontology [59, 60].

## Data Availability

Not applicable.

## Abbreviations

CTO: Cell Tracking Ontology
GFO: General Formal Otology
OBJ: Object
OWL: Ontology Web Language
Q: Quality
QA: Quality Assignment
RDF: Resource description framework
TE: Time Entity
UML: Unified Modeling Language

## Footnotes

1 For the purpose of the current paper the class *Time* is considered as an abstraction of the time parameter and no specific time ontology is assumed.

2 For the sake of simplicity in the examples given here we model all qualities as OWL classes and their values as instances. Clearly, in real life systems different means can be utilized for that purpose, e.g. OWL Enumerated Datatypes or RDF Literals.

3 In a slightly different variant of the pattern a generic *QualityAssignment* class could be used instead of class *ShapeAssignment*. This generalization of handling qualities minimizes the number of classes required in case of diverse qualities. However, it does not influence the discussed patterns as such when it comes to representing the change of quality value over time. Thus, it seems that for the sake of readability the specific properties are more suitable.

4 In case a reification pattern is applied in its generic form with a single generic *QualityAssignment* the amount of T-Box elements for both patterns would be the same.
